# Factors That Shape Eukaryotic tRNAomes: Processing, Modification and Anticodon–Codon Use

**DOI:** 10.3390/biom7010026

**Published:** 2017-03-08

**Authors:** Richard J. Maraia, Aneeshkumar G. Arimbasseri

**Affiliations:** 1 Intramural Research Program, Eunice Kennedy Shriver National Institute of Child Health and Human Development, National Institutes of Health, Bethesda, MD 20892, USA; 2 Commissioned Corps, U.S. Public Health Service, Rockville, MD 20016, USA; 3 Molecular Genetics Laboratory, National Institute of Immunology, Aruna Asaf Ali Marg, New Delhi 110067, India

**Keywords:** anticodon sparing, adenosine 34, inosine 34, tRNA adenosine deaminase, tRNA methyltransferase, La protein RNA chaperone

## Abstract

Transfer RNAs (tRNAs) contain sequence diversity beyond their anticodons and the large variety of nucleotide modifications found in all kingdoms of life. Some modifications stabilize structure and fit in the ribosome whereas those to the anticodon loop modulate messenger RNA (mRNA) decoding activity more directly. The identities of tRNAs with some universal anticodon loop modifications vary among distant and parallel species, likely to accommodate fine tuning for their translation systems. This plasticity in positions 34 (wobble) and 37 is reflected in codon use bias. Here, we review convergent evidence that suggest that expansion of the eukaryotic tRNAome was supported by its dedicated RNA polymerase III transcription system and coupling to the precursor-tRNA chaperone, La protein. We also review aspects of eukaryotic tRNAome evolution involving G34/A34 anticodon-sparing, relation to A34 modification to inosine, biased codon use and regulatory information in the redundancy (synonymous) component of the genetic code. We then review interdependent anticodon loop modifications involving position 37 in eukaryotes. This includes the eukaryote-specific tRNA modification, 3-methylcytidine-32 (m^3^C_32_) and the responsible gene, *TRM140* and homologs which were duplicated and subspecialized for isoacceptor-specific substrates and dependence on i^6^A_37_ or t^6^A_37_. The genetics of tRNA function is relevant to health directly and as disease modifiers.

## 1. Introduction

Transfer RNAs (tRNAs) represent a primordial class of molecules that enabled evolution from an RNA world to a protein coding world. Their principal function in all organisms on earth is to coordinate one of many amino acids with cognate codons in messenger RNA (mRNA)s within the ribosome for concomitant polypeptide synthesis. Concordant with this, there has been striking conservation of overall tRNA structure in the three domains of life, the Bacteria, Archaea and Eukaryota.

A multitude of posttranscriptional nucleotide modifications contribute to the structure and function of tRNAs which are the most densely and complexly modified of all RNAs [1]. While ribosomal and other RNA types carry relatively simple modifications including methylations, dihydrouridine and pseudouridine, tRNAs carry more complex chemical groups [2]. Although there has been much conservation of core modifications, some of these have ‘evolved’ by the acquisition of additional chemical groups added onto the core in a sequential manner by other enzymes [2]. For other modifications, the evolutionary change has been more a matter of extent. Notable here is anticodon wobble position 34, as its modification identity use is associated with a shift in anticodon and codon use patterns on a broad evolutionary scale [3,4,5]. Specifically, the adenosine to inosine modification at position 34 (A34I) is used by bacteria to a very limited extent but its use frequency was significantly expanded in the eukaryotes [4], to apparent near saturation [6]. This was associated with an increase in the number of anticodons used thereby expanding the minimal tRNA pool complexity from 25 in the simplest of the Archaea to an intermediate number in the Bacteria and 46 in the Eukarya, and a cognate shift in codon use [4]. As detailed later, a less extensive shift also pertains to the anticodon-adjacent position 37 and in not so distant eukaryotes including different yeast species. Of course, shifts in this type of tRNA identity-associated modification occur amidst overall sequence changes in accordance with the idea that all tRNAs evolve to achieve a fine-tuned uniform affinity for the ribosome [7,8,9,10]. Accordingly, modifications contribute to the uniqueness of each tRNA.

## 2. Eukaryotic tRNAome Expansions

Presumably, tRNA gene multiplicity has benefits. Analysis of a comprehensive tRNA gene deletion library of *Saccharomyces cerevisiae* revealed that identical tRNA gene sequences at different genomic loci contribute differentially to fitness [11]. In another study, a tRNA gene deletion strain was compensated by mutations of the anticodons of other tRNA genes to match the deleted one [12]. A clever experimental system in the worm, *Caenorhabditis elegans* demonstrated that identical tRNA genes forced to reside in different genomic contexts exhibit different tissue- and time-specific expression patterns [13]. In higher eukaryotes, a brain-specific tRNA gene protects mice from neurodegeneration [14].

Benefits of a more complex tRNAome may be improved fitness, adaptation to translational demands, and greater potential for discriminatory or biased use of synonymous codons [11,15,16,17,18,19]. In model organisms, the cellular amounts of tRNAs, mRNAs and ribosomes set overall translation output based on a population of abundant mRNAs whose codon use generally matches tRNA levels, whereas effects on mRNAs with suboptimally matched codons differ under varying conditions [15,17].

As alluded to above, the copy number of tRNA genes is generally fewer in Bacteria and Archaea relative to Eukarya in which they also expanded to encompass sequence diversity [5,20]. The majority of the sequenced eukaryotes of the *Plasmodia* species all of which are intracellular parasites, contain only 39–57 tRNA genes, and the rest only as many as 78 similar to another eukaryote intracellular parasite, *Leishmania* with 82 tRNA genes similar to the tRNA gene numbers in most bacteria and archaea [20]. Free-living single cell eukaryotes such as yeast contain 200–300 tRNA genes and plants and animals typically contain 400–700 while many contain several to tens of thousands [20,21]. While many mammals including mouse and human and other primates contain from 400 to 700 tRNA genes, pig has 761 and dog 905 [20]. Multiple mammals and other vertebrates contain thousands; the genomic tRNA data base-2 (GtRNAdb2) contains several organisms with tRNA gene counts of 1000–12,000 [20]. It is notable that while the zebrafish genome (*Danio rerio*) harbors 12,000 tRNA genes another fish, *Takifugu rubripes* (puffer fish) contains 588 tRNA genes, more similar to *Homo sapiens* at 506 tRNA genes [20]. For comparison, the relative genome sizes are 400 Mb, 1700 Mb and 3200 Mb of total genomic DNA, for *Takifugu rubripes, Danio rerio, and Homo sapiens,* respectively.

This type and pattern of range suggests a system of dynamic tRNA gene expansion in eukaryotes. Indeed, significant variation has been documented in tRNA gene number among related species and even within individual members of a single species, including yeast and human [22,23,24,25]. Examination of a clade of four *Schizosaccharomyces* species revealed their tRNA gene numbers ranged from 171 to 322 despite very high similarity in the number of other genes, their synteny and architectures, and several other genomic features [23]. Moreover, the tRNA gene number differences among these *Schizosaccharomyces* were accompanied by significant shifts in anticodon use within isoacceptor families [23]. The amoebozoa slime mold, *Dictyostelium discoideum*, represents one of the earliest known branches from the last common ancestor of all free living eukaryotes [26]. *Dictyostelia* can undergo cellular differentiation and many of the genes involved are known to have been inherited by the Metazoa [26]. *D. discoideum* contains 390 tRNA genes, many of which are organized in 41 groups of two and three identical individual tRNA genes suggestive of active waves of duplications [26]. The tRNAomes of higher eukaryotes include sequence diversity within tRNA anticodon families, known as isodecoders [27]. What factors may be involved in the increased number and diversity of these tRNAomes are intriguing questions.

## 3. Eukaryization of tRNA Genes: Monocistrony and Control by a Separate RNA Polymerase

A substantial fraction (up to 75%) of bacterial and archaeal genomic tRNA sequences reside in polycistronic operons with ribosomal RNA (rRNA)s and other tRNAs and/or other RNA types, all of which are transcribed by a singular RNA polymerase (RNAP) (see [28,29]). This organization was revised in two major ways in the Eukarya; the tRNA genes became individual transcription units and under the exclusive control of a dedicated RNAP known as RNAP III, one of the three nuclear RNAPs in all eukaryotes [5]. All known eukaryotes use RNAPs I, II and III for the non-overlapping transcription of the large rRNA, mRNA and tRNA genes respectively as well as some additional RNAs (higher plants also have IV and V which are closely related to RNAP II [30,31]). RNAP III contains seventeen integral subunits [30,32,33]. In the yeast model organism *S. cerevisiae,* RNAP III controls transcription of genes that encode six different types of small structural RNAs; 5S rRNA, U6 spliceosomal snRNA, scR1 small cytoplasmic RNA of the signal recognition particle (SRP), RPR1 RNase P RNA, the snr52 small nucleolar RNA, and all the different tRNAs. All but the tRNAs are single sequence genes that produce structured RNAs that undergo relatively little processing before becoming a stable part of their corresponding multisubunit ribonucleoproteins (RNPs); 5S rRNA is produced from 50 to 150 gene copies [34] that express identical RNA. By contrast, 275 genes encode the tRNAs in *S. cerevisiae* and these encompass a spectrum of sequence and length diversity including many with introns and/or variable arms in addition to their variable 5′-leaders and 3′-trailers, and undergo multiple processing and modification steps [35,36].

In addition to the multisubunit transcription factors (TF), TFIIIC and TFIIIB that direct transcription initiation, three of the RNAP III-specific subunits contribute to its termination–reinitiation activities [37,38] that are critical for high output production of total tRNAs to levels that are 10–12 fold higher than ribosomes [39]. One of these subunits, Rpc11, is required for 3′ cleavage of elongating transcripts whose synthesis becomes paused or arrested on the transcribed gene template [40,41]. While similar factors/activities exist for RNAP I, RNAP II, bacterial and archaeal RNAPs, Rpc11 is the only one that is essential [40]. In bacteria, collision of the replication machinery with an arrested RNAP can cause double strand DNA breaks [42]. It is known that in yeast, RNAP III transcription factors interact genetically with the DNA replication machinery [43,44], providing a plausible link to the apparent propensity for amplification of tRNA genes in eukaryotes. What factors may be involved in the other aspects of eukaryotic tRNAome expansion?

## 4. The pre-tRNA Chaperone, La Protein, Is Directly Targeted to RNAP III Transcripts

The deepest rooted eukaryotes contain La and RNAP III and therefore would appear to have arisen concurrently [45]. The RNAP III transcription termination mechanism is unique among eukaryotic RNAPs and is part of its specialization for high efficiency production of short transcripts [46,47]. A fundamental component of this mechanism is the termination signal, a short tract of ≥5 T residues in the non-template DNA strand found at the 3′ ends of RNAP-transcribed genes [48,49,50,51]. Upon encountering the oligo(T) termination signals of individual tRNA genes, RNAP III synthesizes the matching oligo(U) 3′-OH motif [41,50], which is the high affinity binding target for La, the first protein known to interact with nascent pre-tRNAs (see section 2.11 in [52]). La is an abundant protein (ca. 2 × 10^7^ molecules/HeLa cell nucleus) that is mostly nucleoplasmic but also found at RNAP III-transcribed gene loci in vivo, and part of a RNAP III holoenzyme [53,54]. Length of the 3′ oligo(U) motif is a principal and functional determinant of a pre-tRNA’s competitive affinity for La protein [55,56]. Depending on oligo(U) length and other features [57], a nascent pre-tRNA may proceed through a course of alternate intranuclear processing pathways that include multiple modifications [58,59,60] that contribute to the unique sequence and modification identity of a particular tRNA [36,59,61].

La proteins from yeast and human exhibit two activities, RNA oligo(U) 3′ end binding with consequent protection from 3′ exonucleolytic digestion, and RNA chaperone activity [62,63]. This chaperone activity can assist the folding of those pre-tRNAs that are susceptible to misfolding, and it does so by using an RNA binding surface that is distinct from its oligo(U) 3′ OH-interacting surface [57,63,64,65,66]. While La binds to the oligo(U) ends of all types of nascent RNAP III transcripts and can protect them from untimely 3′ end-mediated intranuclear degradation, an increasing number of studies indicate that its RNA chaperone activity is most if not exclusively beneficial to the pre-tRNAs [64,67]. Large scale genome-wide screens uncovered several tRNAs to which point substitutions cause cells to require La for growth [64,65], see [67,68,69]. We know of no genes encoding non-tRNA transcripts that were uncovered by this approach although mutations to proteins involved in U6 snRNP assembly were identified [70,71]. La can protect scR1 and other non-tRNA transcripts from untimely decay due to 3′ oligo(U) binding [72,73]. Nascent pre-5S rRNA is transiently bound by La and although it undergoes little if any processing, its maturation would appear to be largely independent of La (e.g., see [74,75]).

The complexity of the nascent transcripts of a eukaryotic tRNAome, including 5′-leaders, 3′-trailers, introns and variable extra arms, is large [27]. It is not surprising that some of the pre-tRNAs would exhibit misfolding into non-tRNA-like structures. La’s function for pre-tRNAs extends beyond 3′ end protection, as it also serves to prevent the misfolding of those sequences with propensity to form alternate structures [64,65,67,68]. In some cases, susceptible pre-tRNAs will succumb to nuclear surveillance-mediated decay in the absence of La [57,63,69]. Some tRNA structures that are weakened in a single base pair, e.g., G:C✱G:U, in the anticodon stem, also require La for correct folding [64], see [67,69]. The RNA chaperone activity of La was demonstrated to be important at low temperature, as La-deleted cells exhibit inefficient charging of one or more tRNAs, reflecting suboptimal structure [64]. 3′ oligo(U) length varies among pre-tRNAs, even from a single tRNA gene locus, and this is a functional determinant for structurally challenged pre-tRNAs in their competition for the La-dependent pathway of maturation [55], reviewed in [60]. In summary, the pre-tRNAs are more numerous and structurally challenged as a group than other RNAP III transcripts, and the cumulative data indicate that La is targeted to and most effectively assists their maturation. Because La exhibits general RNA chaperone activity [66,76] it would appear to be well suited to assist various individual pre-tRNAs that comprise the complex population [57,59,63,64,65].

## 5. Nuclear pre-tRNA Modification Enzymes Can Also Avert pre-tRNA Misfolding

As noted above, some eukaryotic pre-tRNAs are susceptible to misfolding and nuclear decay and some form tRNA structures that are inefficiently charged in the absence of La and/or certain nuclear modifications [58,59], see [60,64,77]. Several modification enzymes have been identified that function similarly to La toward stabilization of otherwise susceptible pre-tRNAs thereby assisting their conversion to functional tRNAs (also see [58,59]). When any of these enzymes are lacking, either alone or usually together with La, certain pre-tRNAs become susceptible to degradation and/or dysfunction. These are Trm1, Trm2, Trm3, Trm6, Pus3, Pus4; of the five of these whose subcellular localization have been determined, all were found in large part to be nuclear [58,59,78,79,80,81]. Trm2 appears to have pre-tRNA chaperone activity independent of its catalytic activity [78]. Redundancy of pre-tRNA chaperone activity by La and tRNA-interacting enzymes would appear to extend to some tRNA synthetases as well [59], not inconsistent with a quality control function of nuclear tRNA charging [82].

## 6. Factors That Would Support Eukaryal tRNAome Expansions

It is now appreciated that in addition to the direct involvement of assisting the folding of their ligands, chaperones can also serve an evolutionary function by ‘buffering’ mutations in their substrates so that otherwise ineffective isoforms can become useful [83,84]. Thus, it is plausible that La and other pre-tRNA chaperones could provide this function. As noted above, the gene duplication machinery itself which was accompanied by increased diversity, was critical to the expansion of eukaryotic tRNAomes. However, by mechanistically linking La with its chaperone activity to nascent pre-tRNAs via sequence specificity for the oligo(U) 3′-OH products of RNAP III, all eukaryotic tRNA genes would be well equipped. With La at their end, any duplicated, amplified or mutated tRNA gene sequences that might otherwise produce misfolded or suboptimal tRNA structures would have an increased chance to yield functional tRNAs.

Larger, more diverse and more readily flexible tRNAomes might afford better adaptation to translational demands, that may also include greater potential for biased codon use. The apparent advance of dedicated control over individual tRNA genes and their duplication/amplification as such, with the concurrent appearance of the associated La protein pre-tRNA chaperone may have facilitated expansions of eukaryal tRNAomes with greater diversity of sequence, biased codon use, and adaptation to translational demands (see [85]).

## 7. Eukaryotic tRNA Anticodon-Sparing

**Figure 1:**
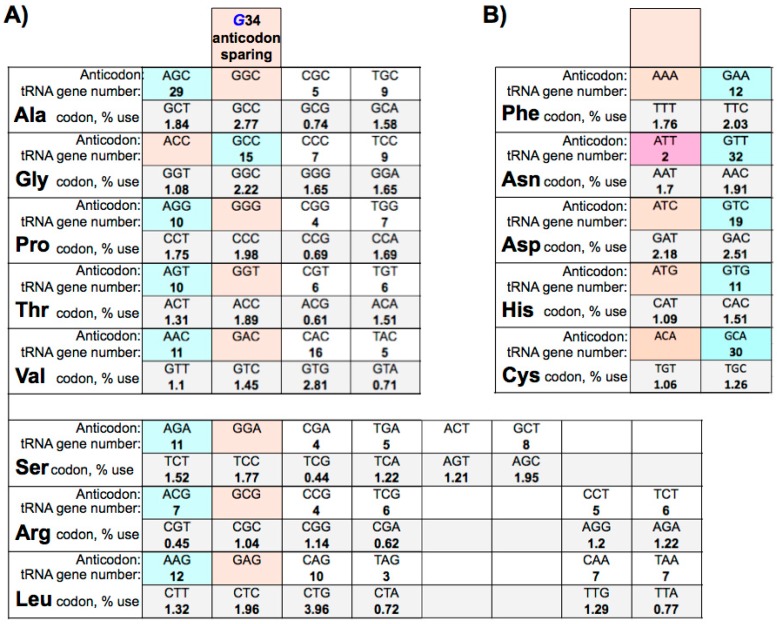
Examples of G34 and A34 anticodon-sparing in the human tRNAome. (**A**) G34 anticodon sparing predominates in the four-box and six-box codon sets. The tRNA gene numbers are listed for each anticodon. Blue shaded rectangles contain numerous tRNA genes for the same A34 anticodon and salmon colored rectangles indicate absence of any tRNA gene encoding a G34 anticodon for the same amino acid. Note that in each case where the A34 anticodon is used its A34 is converted to inosine and the overall codon % use is higher for the wobble codon. Non-colored rectangles show tRNA gene copy numbers for anticodons with C or T at the 34 position; (**B**) A34 anticodon sparing predominates in the two-box codon sets, only some of which are shown here. Blue shaded rectangles contain numerous tRNA genes for the same G34 anticodon and salmon colored rectangles indicate absence of any tRNA gene encoding a A34 anticodon for the same amino acid. The pink colored rectangle reflects a case where two genes with ATT codons exist (see text). More examples of these so-called exceptions to otherwise forbidden anticodon genes are provided in Figure 2. This figure is a partial summary of a tRNAscan-SE analysis of *Homo sapiens* (hg19 - NCBI Build 37.1 Feb 2009 found online at http://lowelab.ucsc.edu/GtRNAdb/Hsapi19/).

Extensive analyses have revealed three major ‘anticodon-sparing strategies’ that collectively provide organisms the means to decipher genetic code information with an economy of tRNA anticodons required to decode all of the sense codons [5]. All three major anticodon-sparing strategies have been well supported by subsequent analyses of increasingly larger genome data sets [3,86]. The A34 and G34 anticodon-sparing strategy is considered as one of the three major strategies and is the one most relevant to the eutRNAomes to be discussed here. This strategy arose from data that show that genomes that contain tRNA genes encoding A at 34 do not simultaneously contain isoacceptor tRNA genes encoding G34 (and identical at the other anticodon positions) [3,5]. Likewise, genomes that contain tRNA genes encoding G34 do not also contain isoacceptor tRNA genes encoding A34 [3,5]. Sparing the need for G34 anticodons is enabled by the conversion of A34 to I34 in transcribed tRNAs which can decode both of their cognate U ending and C ending codons [3,87]. G34 anticodon-sparing is extensive in eukaryotes as it usually occurs in seven of the eight boxes with four- and six- codons, with the exceptional disparity of Gly (below and Figure 1) apparently serving to economize on the number of tRNA anticodons [3].

Whereas A34I modification supports G34-sparing, the substrate recognition specificity of the A34I enzyme, known as heterodimeric adenosine deaminase acting on tRNA A34 (hetADAT), can account for disparity in G34 sparing. It was shown that yeast hetADAT can efficiently modify several natural tRNA-A34 substrates (and synthetic test substrates) if they contain a purine at position 35 [88,89]. As noted above, of the five tRNA isoacceptor families for the four-codon box sets, the only one that uses G34 tRNA genes rather than A34 genes in *Saccharomyces cerevisiae, Schizosaccharomyces pombe*, *Caenorhabditis elegans, Drosophila melanogaster*, humans and others [20], is Gly (see Figure 1 for example). The basis for this profound exception was recently investigated and revealed that the structural context of the anticodon loop appears to be a highly discriminatory determinant of substrate specificity of hetADAT [6]. This work goes beyond the prior noted exception to the position 35 specificity for purine that is exhibited by tRNA^Arg^ACG which is a substrate for hetADAT [88,89] which allow tRNA^Arg^ACG genes to predominate in eukaryotes which exhibit G34 anticodon-sparing [20]. Using biochemical approaches with mutagenesis and domain swapping as well as molecular dynamics simulations, Saint-Léger et. al., showed that inability of hetADAT to modify tRNA^Gly^ACC is not due to a specific sequence but rather to the structural context of its anticodon [6]. This led to the conclusion that structural features of the tRNA^Gly^ACC anticodon loop would appear to be incompatible with a functional A34 and this provides explanation for why tRNA^Gly^ACC genes were not enriched in eukaryotes after emergence of hetADAT [6]. We will return to address biological aspects of G34 anticodon-sparing in a later section.

## 8. Adenosine 34 Anticodon-Sparing

The extent of A34 anticodon-sparing in eukaryotes can be appreciated by examining the case provided by the appropriate human tRNA gene profile in Figure 1B. Similar patterns are found in other eukaryotes although the exact numbers of the tRNA genes involved varies widely. However, the strategy for A34 anticodon-sparing in eukaryotes, goes beyond economy per se as it serves to avoid deleterious miscoding [3]. This stems from conversion of A34 to I34 in eukaryote tRNAs. As previously noted [3], the lack of A34-containing tRNAs in the two-synonymous codon boxes (e.g., Figure 1B), and use of the G34 anticodons instead, results from the avoidance of miscoding by a wobble base, A34I of purine-ending codons in other boxes [3]. Thus, A34-tRNAs in the two-synonymous codon boxes would be expected to be deleterious in eukaryotes and should be avoided. For example, if tRNA^Phe^AAA existed it might be converted to tRNA^Phe^IAA and wobble decode Leu UUA codons, and if tRNA^His^ATG existed it might be converted to tRNA^His^ITG and wobble decode CAA Gln codons, and so on for the other A34-tRNAs in the other two-synonymous codon boxes. In the cases of Tyr and Cys, the A34I-tRNAs would suppress stop codons. Accordingly, as an illustration, the human tRNAome contains 12, 11 and 30 tRNA G34 anticodon genes for Phe, His and Cys respectively but no tRNA A34 anticodon genes for these [20].

Three apparent mechanisms prevent the potential for miscoding by A34-tRNAs in the two-synonymous codon boxes, the restrictive substrate specificity of the hetADATs, restrictive wybutosine-37 (yW37) formation on tRNA^Phe^GAA but not on tRNA^Phe^AAA, and A34 anticodon-sparing. Of the seven two-synonymous codon boxes with the A34/G34 arrangement, six of their A34-tRNAs if they were to exist, would have the hetADAT-unfavored pyrimidine at 35, the other would be tRNA^Phe^AAA if it was to exist. Although tRNA^Phe^AAA would appear to be a good substrate for hetADAT by the purine-35 criterion [89] and by experimentation using synthetic substrates [88], this may not be problematic for miscoding because it would not be expected to be properly modified to yW on position 37. As detailed below, expression of eukaryotic tRNA^Phe^ is exceptional as it acquires the unique yW37 modification and in yeast requires retrograde cytoplasmic–nuclear transport for maturation [90]. While the first step in yW37 formation, m^1^G_37_ modification occurs independent of the tRNA^Phe^ anticodon sequence, later step(s) exhibit strict requirement for a GAA anticodon; a tRNA^Phe^AAA did not support yW37 formation after injection into *Xenopus* oocytes [91].

## 9. Eukaryotic tRNA Guanine 34 Anticodon-Sparing

While mechanisms exist to avoid the potential for deleterious miscoding from expression of A34-tRNA genes of the two-synonymous codon boxes if they were to exist, no apparent deleterious miscoding would be expected from G34-tRNA representatives of the four- and six- codon boxes (according to known wobble rules [92]). Thus, G34 anticodon-sparing among eukaryotic tRNAomes might be due solely to economy. However, the pervasive persistence of G34 anticodon-sparing in higher eukaryotes, including those with moderately large tRNAomes (humans) might suggest selective pressure against these G34-tRNAs. A typical example concordant with G34 anticodon-sparing is provided by human tRNA Ala, Ser and Arg anticodons; 29, 11 and 7 genes exist for tRNAs Ala, Ser and Arg anticodons AGC, AGA and ACG respectively, but there are no genes for tRNAs Ala, Ser and Arg anticodons GGC, GGA and GCG; a similar pattern is found in budding and fission yeasts, and *D. melanogaster,* no G34 genes [20]. This degree of evolutionary exclusion, including in human for which there are 29 tRNA^Ala^AGC genes but no tRNA^Ala^GGC, would suggest a selective pressure greater than economy.

What might be a candidate selective pressure beyond economy of number of tRNA genes? Having the A34-tRNA but not the G34-tRNA, would force all synonymous codons with C in the wobble position to be dependent on the A34I modification. This may potentially be used to the differential benefit or disadvantage of sets of mRNAs that are biased with one or the other of the synonymous codon. A system of biased splitting of such synonymous codons among mRNAs may have provided selection pressure that contributed to the tRNA gene exclusion that accounts for G34 anticodon-sparing in eukaryotes. The ‘regulatory’ or other basis on which the corresponding mRNAs would be grouped in different eukaryotes would be flexible, or species-specific, as part of a system of secondary information derived from the redundancy component of the genetic code [18]. This system makes use of the over-representation and biased use of one or the other member of a synonymous codon pair, among functionally related mRNAs, that are responsive to a specific tRNA anticodon wobble base modification, for example as demonstrated experimentally [93].

Although the proposal that G34 anticodon-sparing may have evolved to preserve the ability to utilize potential secondary information in the redundancy of the code has not yet been supported by experimental data, it is consistent with results from gene ontology (GO) pathway/category analysis of codons sensitive to A34I modification [87]. This revealed a number of remarkable findings: (1) significant grouping of mRNAs into those enriched or depleted of A34I-sensitive codons; those mRNAs enriched exhibit 30–1000% more GOs than those depleted of A34I-sensitive codons in the four species examined; (2) the number of GO categories increase with biological complexity of the species; and (3) the vast majority of the GOs found for A34I-enriched proteins are species-specific for each species. The analysis was performed in a way that provides evidence of adaptation of A34I-sensitive codon usage by functionally related mRNAs, i.e., acting in similar processes [87], and is consistent with their recruitment so as to preserve the potential for utilizing secondary information in the redundancy of the code. However, as the analyses were done, they indicate that A34I-sensitive codons are generally enriched in functionally related mRNAs but do not distinguish among the cognate vs. wobble codons. The proposal that G34 anticodon-sparing may have evolved and/or was selected to preserve the potential for secondary information use would be significantly strengthened if the cognate vs. wobble codons serviced by A34I-tRNAs were themselves further divided among distinguishable sets of mRNAs. As reviewed in a later section, emerging evidence is beginning to suggest this may be the case. Specifically, modulation of tRNA^Ser^AGA nutrition-dependent A34I modification, coupled to analysis of the disparity of UCU and UCC codons in functionally-related mRNAs in fission yeast, supports this proposal [94].

## 10. Large tRNAomes Disregard Guanine 34 Anticodon-Sparing and Adenosine 34 tRNA Genes

**Figure 2:**
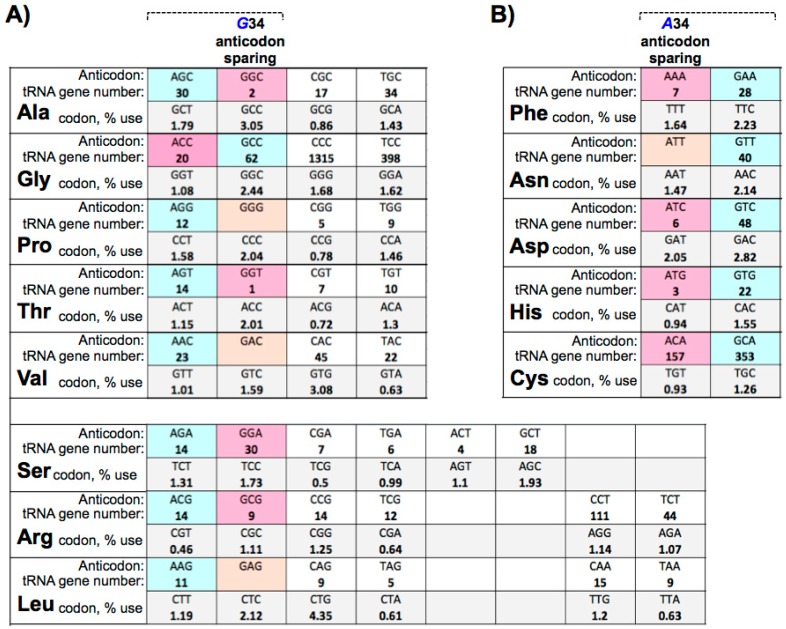
Example of disregard for G34 anticodon-sparing and A34 tRNA genes by the large tRNAome of *Bos taurus* (cow). The scheme is organized as for Figure 1 with same color code. This figure is a partial summary of the total number of tRNA genes which is 4161 obtained by tRNAscan-SE analysis (Baylor release Btau_4.0, October 2007) as can be found at http://lowelab.ucsc.edu/GtRNAdb/Btaur/.

Examination of some of the large eutRNAomes reveal multiple exceptions to A34 and G34 anticodon-sparing in cat, zebrafish, cow and others. The overall extent is variable although in several organisms multiple anticodon boxes are involved [20]. In the yeast which have 200–300 tRNA genes, there are usually no tRNA genes for A34 anticodons in the two box codon sets, e.g., corresponding to Figure 1B. However, this begins to break down at a low level as the tRNAome size increases such as in human, for Asn (Figure 1B); in this case 32 tRNA^Asn^GTT genes dominate over two tRNA^Asn^ATT genes. Examples of large tRNAomes that disregard G34 anticodon-sparing and A34 tRNA genes are provided in Figure 2. A striking example is *Bos taurus* (cow) in which the total number of A34 and G34 anticodon-sparing tRNA genes comprises nearly 6% of the tRNAome. In this case, 14 tRNA^Ser^AGA genes coexist with 30 tRNA^Ser^GGA genes, 14 tRNA^Arg^ACG genes coexist with nine tRNA^Arg^GCG genes, 20 tRNA^Gly^ACC genes coexist with 62 tRNA^Gly^GCC genes, and 157 tRNA^Cys^ACA ‘prohibited’ genes coexist with 353 tRNA^Cys^GCA genes (Figure 2) [20]. The prohibited genes are not limited to those that might result in suppression of UGA stop codons as 10 of the tRNA^Tyr^ genes bear A34 and if productive of functional tRNA would suppress UAA stop codons. Most strikingly, between 13% and 20% of the tRNA genes for each of the Phe, Asp and His boxes in the cow tRNAome encode A34 [20]. Although the tRNAscanSE COVE and other scores for these vary, some appear to have potential as tRNAs [20] and therefore for miscoding.

The significance of the exceptions to G34 anticodon-sparing and the numerous predicted A34 prohibited genes in the large eutRNAomes is unknown. A consideration is that they are pseudogenes although the tRNAscanSE algorithm used to predict them distinguishes real from pseudogenes [20]. Comparison of tRNAscanSE scores for secondary structure and other parameters such as consensus sequence promoter elements for some of the A34 and G34 isoacceptor pairs reveals substantial overlap suggesting the possibility that both classes may produce functional tRNA [20]. In other cases, one anticodon family of an isoacceptor pair yield lower scores than the other, yet they were not counted as pseudogenes [20]. Whatever process led to their amplification it may have preserved the potential for expression. A tRNA^Ala^CGC-derived SINE gave rise to the BC1 tRNA-like gene in rodents [95,96,97]. Thus, regardless of their origin or derivation, assays for expression and function for these annotated tRNA genes should be forthcoming and they should be noted because they are interesting exceptions and previously renowned as nonexistent and as never occurring [3,86,87,98]. These observations raise questions. Is their expression restricted to specific tissue and/or developmental or stress-related programs and if so is there cognate codon bias? Are the A34 tRNA genes expressed and do the pre-tRNAs undergo A34I modification as would be expected [99], and do they produce cytosolic tRNAs? Do they produce tRNA fragments?

## 11. tRNA Adenosine 34 to Inosine May Be Keyed to Differential Synonymous Codon Splitting

As noted earlier, a major evolutionary shift occurred in the extent of A34I modification, accompanied by increased tRNAome complexity. Specifically, eukaryotes ‘evolved’ more A34 anticodon genes than bacteria or archaea, along with a cognate shift in codon use [4]. This represents a tRNA wobble modification keyed to a shift in codon use on an evolutionary scale. The numbers of tRNAs and codons involved were substantial [4]. A34I is essential for yeast cell growth and survival [100].

Recent quantification by tRNA-HySeq revealed that unlike several other modifications whose stoichiometry varies widely among individual yeast tRNAs, nearly all A34 was efficiently converted to I (ca. 90%) on all of the A34-containing tRNAs [94], consistent with known stoichiometry [101]. However, as will be reviewed below for U34 and C34 modifications which can be used for dynamic control of translation, e.g., in stress responses, A34I has not been linked to dynamic translational control although this remains an open possibility [87]. A benefit of tRNA-Seq methods that monitor A34I efficiency as misincorporation [99] is that they can do so for individual tRNAs and therefore allow detection of tRNA-specific variances [94]. Toward the possibility that alterations in A34I levels may be associated with dynamic control, we note that its modification stoichiometry was reduced from 90% to 65% for *S. pombe* tRNA^Ser^AGA in rich vs minimal media growth conditions [94]. The basis of the specificity and mechanism of decreased A34I efficiency for tRNA^Ser^AGA are unknown.

**Figure 3:**
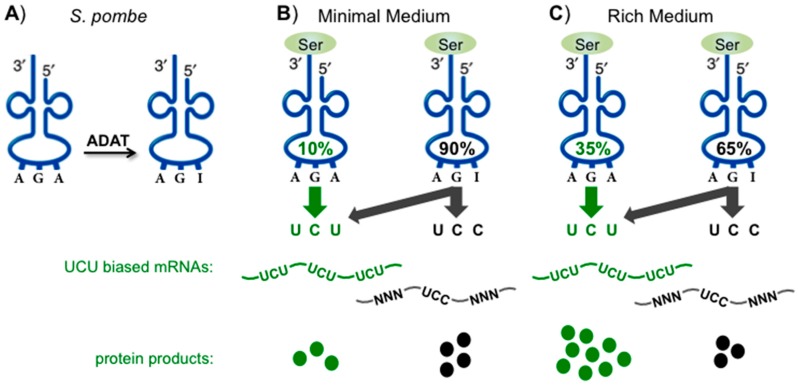
Differentially modified adenosine-34 to inosine-34 (I34) in tRNA^Ser^A/IGA may be keyed to synonymous codon splitting in the fission yeast, *Schizosaccharomyces pombe*. (**A**) The enzyme adenosine deaminase acting on tRNA (ADAT) converts A34 in tRNAs to I34, shown here for tRNA^Ser^A/IGA; (**B**) In minimal medium, the relative amounts of tRNA^Ser^AGA and tRNA^Ser^IGA are 10% and 90% as indicated in the tRNA cartoon [94]. The downward vertical arrows reflect relative efficacy of the AGA and IGA anticodons for UCU and UCC codons; IGA can decode both UCU and UCC codons whereas AGA would more readily decode the UCU over the UCC codon according to wobble rules [92]; (**C**) Rich media produces faster growth and requires high levels of protein synthesis, including production of ribosomal proteins whose mRNAs are highly abundant and are enriched/biased in UCU codons and lack UCC codons (see text). As the percentage of unmodified tRNA^Ser^AGA increases in rich media those tRNAs are directed to the UCU-biased mRNAs because without I34 they cannot readily decode the UCC containing mRNAs (see text).

The specificity and degree of rich media-induced decrease in A34I conversion for tRNA^Ser^AGA is noteworthy in its own right. However, together with analysis of cognate (UCU) vs wobble codon (UCC) use by tRNA^Ser^A/IGA, the observed modulation of A34I may provide evidence of function in making use of information in the genetic code redundancy and further rationale for tRNA-A34 gene exclusion from eukaryotes. Multiple mechanisms are used by cells to efficiently make ribosomes and other components of the translational machinery during fast growth in rich media. It was noted that rich media-mediated modulation of tRNA^Ser^IGA might be linked to the UCU and UCC codon frequency in ribosomal protein mRNAs in *S. pombe* [94]. tRNA^Ser^IGA must decode UCU and UCC codons as there is no tRNA-G34 anticodon (G34 anticodon-sparing) in the *S. pombe* tRNAome. The UCU codon is used 2.5-fold more frequently than the UCC codon in the overall *S. pombe* transcriptome [20], but the ratios differ four-fold in high vs. low-expression mRNAs ([102] and references therein). As the fraction of tRNA^Ser^IGA decreases in rich media, the relative amount of tRNA^Ser^AGA increases, and UCU codons would be at a relative advantage over UCC codons. The mRNAs with an excess of UCU over UCC codons would benefit relative to those with excess of UCC codons which would be at a disadvantage because there would be less tRNA^Ser^IGA to decode them while the UCU codon-biased mRNAs could still be decoded by tRNA^Ser^AGA (Figure 3). In *S. pombe* the advantage would be for efficient translation of ribosomal protein mRNAs as expected for fast growth (below).

Table 1 shows the disparity of use of the two codons, UCU and UCC that are read by tRNA^Ser^IGA in *S. pombe* mRNAs. Table 1 summarizes results of analysis of 5012 gene mRNAs sorted by their UCU:UCC ratio and total abundance [103] using previously determined quantitation of transcript copy number [104]. Scores of 1.0, 0.5 and 0 represent mRNAs with all UCU and no UCC codons, an equal number of UCU and UCU codons and, all UCC and no UCU codons, respectively. Twenty-nine gene mRNAs contain neither UCU nor UCC codons (not shown). Table 1 shows only 41 mRNA sets from each of the three sets. Only the set with over-enrichment of UCU codons shows significant enrichment of GO terms, and a term is structural constituent of the ribosome. This reflects that 22 of the top 41 mRNAs in this category encode ribosomal proteins. The bottom and middle sets yielded no GO terms (Table 1). The number of mRNAs with a score of 1.0 was 371 but we limited the analysis to the top 41 because the number of mRNAs with a score of 0 was 41 (presence of UCU but no UCC codons). However, when this set of 371 mRNAs was analyzed it yielded similar results, with ‘structural constituent of the ribosome’ as a top enriched term, and included the mRNAs for 37 ribosomal subunits as among those with UCU codons but no UCC codons (not shown). This analysis shows significant splitting of synonymous Ser codons that are read by tRNA^Ser^IGA and in a manner concordant with a decrease in A34I conversion in rich growth media so as to favor ribosome biogenesis. The potential use of I34-mediated synonymous codon splitting toward translational regulation of cognate synonymous codon-biased mRNAs is schematically depicted in Figure 3.

## 12. Targeting Sensitive Synonymous Codons for Cognate Specific Response

As noted above the redundancy component of the genetic code can harbor various types of secondary information (i.e., in addition to that dictating the primary amino acid sequence of a polypeptide) which can include effects on pre-mRNA splicing, mRNA folding which itself can manifest as multiple effects, ribosome pausing with potential effects on polypeptide folding, and others [18,19,98]. Another type of secondary information is that which can be used for feedback regulation. An example is found in the control of amino acid biosynthesis in response to changes in nutritional status in *E. coli* [105] These mRNAs regulate the production of their encoded amino acid biosynthetic enzymes by use of upstream synonymous codons whose cognate tRNA is the most sensitive to aminoacylation under starvation for the specified amino acid. This enables translation of these mRNAs under the nutrient conditions as required [105]. mRNAs encoding different metabolic enzymes employ a similar strategy by preferential use of their corresponding synonymous codon whose tRNA is most sensitive to the amino acid cognate to the biosynthetic pathway at hand [105]. This system reflects an elegant utility of this type of secondary genetic code information. However, it is a complex system that relies on the physical connectivity of transcription and translation in bacteria [106]. These examples are codon-specific to individual mRNAs. Other exploitations or ‘lifting’ of the genetic code degeneracy is by splitting codon families into hierarchies of isoacceptor tRNAs that differentially compete for amino acylation in *E. coli* [17].

Below, we review an expanded, more organized use of secondary information involving functionally-related mRNAs that share patterns of synonymous codons as a means of translation control, whose coordination is via tRNA wobble modification. As detailed in the next section, this type of secondary code information was first described for yeast, and was followed by additional examples involving separate wobble modifications and cognate groups of mRNAs. This was recently extended to Bacteria, in the *Mycobacterium tuberculosis* surrogate, *Mycobacterium bovis* during hypoxia-induced non-replicating persistence [107].

## 13. Biased Codons Keyed to tRNA Wobble Modification for Programmed Stress Response

As noted, anticodon wobble modifications can alter base pairing properties and extend the mRNA decoding activity of tRNAs [92]. Indeed, the anticodon wobble is the most diversely modified nucleotide on eukaryotic tRNAs [3,35,36,101,108]. Some of these modifications enhance while some may restrict wobble pairing [109]. A range of approaches indicate that for tRNAs that decode more than one synonymous codon, some anticodon wobble modifications can promote decoding of one over the other codon (see [110]). Activity for anticodon wobble base modifications in distinguishing codons have been demonstrated in living yeast cells [111].

The breakthrough in this area came from deep analysis indicating that translation of a functionally-related group of mRNAs share a pattern of biased synonymous codons that are complemented by an anticodon wobble modification of the cognate tRNAs, as part of a programmed stress response [93]. This indicated that tRNA anticodon modifications can be used dynamically, in real time, to distinguish synonymous codons as part of a stress response involving multiple mRNAs that share the same general bias of receptive codons. Specifically, *S. cerevisiae* DNA damage response genes are enriched for Arg(AGA) and Glu(GAA) codons relative to their Arg(AGG) and Glu(GAG) codons [93]. The tRNAs ^Arg^UCU and ^Glu^UUC that decode these codons are specific substrates of Trm9, the methyltransferase that generates mcm^5^U34 from cm^5^U34 [112]. Begley et al. found large biases of AGA and GAA codons with ratios of 41:0 AGA:AGG and 91:1 GAA:GAG in mRNAs encoding for some ribosomal proteins, the translation elongation factor Yef3p, and a family of ribonucleotide reductases (Rnr1p–Rnr4p) involved in dNTP synthesis [93]. The Yef3p, Rnr1p and Rnr3p proteins were less abundant in strains lacking Trm9 (*trm9*-∆) relative to wild-type cells, despite similar mRNA levels [93] reflecting dependence on *TRM9* for wild type high levels. Trm9-mediated U34 modification of tRNA^Arg^UCU promotes efficient translation of the cognate AGA codon while restricting wobble to the synonymous AGG codon [113,114].

Furthering this is research showing translation of UUG codon-enriched oxidative stress responsive mRNAs promoted by the wobble modification 5-methylcytosine (m^5^C_34_) of tRNA^Leu^CAA by Trm4 in yeast, without which cells are hypersensitive to hydrogen peroxide [115,116]. Another tRNA anticodon uridine wobble modification, mediated by Sin3/Elp3 in fission yeast, that is linked to translation of an mRNA set with biased synonymous codons, also followed [117].

This type of biased codon use coordinated with anticodon wobble base modification as a means of control was recently uncovered in the Bacteria, *M. bovis*, a model of the pathogen *Mycobacterium tuberculosis*. During hypoxia-induced persistence, a phenomenon that occurs during tuberculosis granulomas formation, cmo^5^U modification of tRNA^Thr^UGU increases to aid translation of mRNAs enriched with the cognate codon [107].

**Figure 4:**
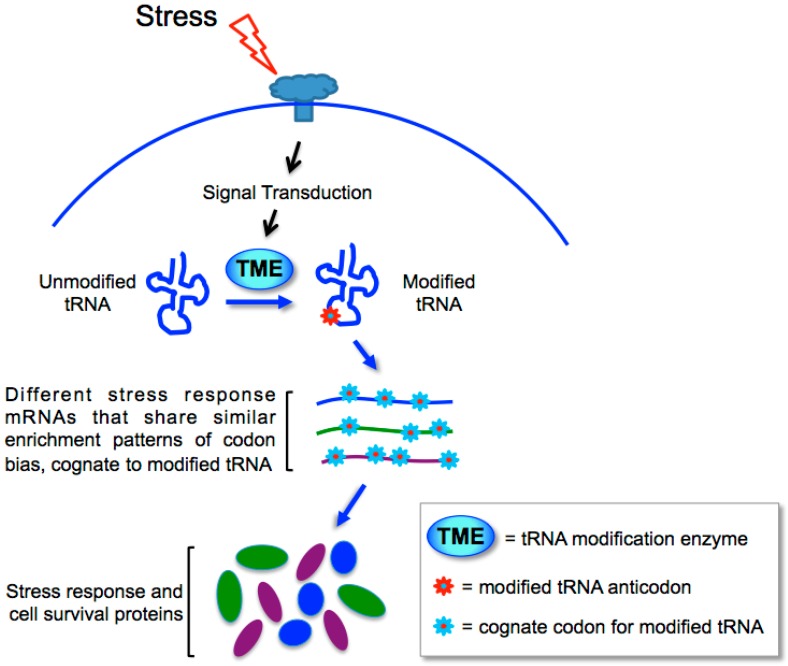
General model for how tRNA modifications can be keyed to a cellular stress response. Upon stress, the modification efficiency of a particular tRNA, usually at the wobble nucleotide, is increased (see text and [118]). These modifications favor a shift in the efficiency of translation of functionally-related mRNAs that are enriched in the cognate codons, producing proteins that contribute to an appropriate response.

A general schematic model for how tRNA anticodon modifications are keyed to the translation of cognate codon-biased mRNAs is depicted in the cartoon in Figure 4. In this model, the system is keyed through tRNA modification enzymes and tRNA modification-dependent preferential translation of mRNAs that confer a stress response (see [118]).

## 14. Deriving Pliable ‘Secondary’ Information from the Redundancy of the Genetic Code

Multiple collective examples from yeast and bacteria [93,107,115,116,117] provide compelling cohesive evidence to suggest that the redundancy component of the genetic code is widely used as a means of secondary information involving wobble-dependent, coordinated translation of functionally-related mRNAs [18,118,119,120]. This system comprises one type of secondary information in the redundancy component of the code [16,18,19]. This type of secondary or auxiliary genetic code information consists of three constituents, mRNAs with shared patterns of synonymous codon bias, a tRNAome that complements the cognate codon usage, and tRNA anticodon modification activities that distinguish synonymous decoding [18] (Figure 4). Unlike the primary information in the genetic code which is fixed as each sense codon is assigned a specific amino acid in any particular organism, secondary information is flexible, such that the functional pathways of the mRNAs assigned to a biased synonymous codon can differ in different species (or tissues perhaps), as well as the number of different synonymous codons involved in the mRNA sets, and the hierarchy of sensitivities of the individual mRNAs within the sets. The potential amount and complexity of information can be enormous. While all organisms use the primary genetic code information in the more or less same ‘universal’ way to encode the amino acid sequence of polypeptides [16,19], they can use this type of secondary information in the code in species-specific ways.

## 15. Differential Presence and Secondary Code Use of t^6^A_37_ and i^6^A_37_ Modifications

The decoding performance of different anticodons is enhanced by the identities of their position 37 and adjacent nucleotides [121]. The nucleotide adjacent to the anticodon 3′ end is position 37, one of the most diversely modified nucleotides in tRNA. In bacteria, most if not all of the tRNAs that read codons starting with U or A make a weak anticodon:codon base pair with the corresponding A36 or U36 anticodon nucleotide of the cognate tRNA, and have a large bulky modification in position 37, either threonlycarbomyladenosine-37 (t^6^A_37_) or isopentenyl-*N^6^*-adenosine (i^6^A)-37 (or their derivatives) to stabilize it [122].

t^6^A_37_ and/or its derivative is found on many tRNAs in all three domains of life. In some species, t^6^A_37_ exists as is whereas in others it is in a cyclic form, ct^6^A_37_ (see [101,123]) or a hypermodified form thereof, hereafter referred to collectively for simplicity as t^6^A_37_ [124,125]. As t^6^A_37_ is found almost without exception on all tRNAs that decode the sixteen ANN codons, it is a most pervasive anticodon loop modification (see [101,123,126]).

**Figure 5:**
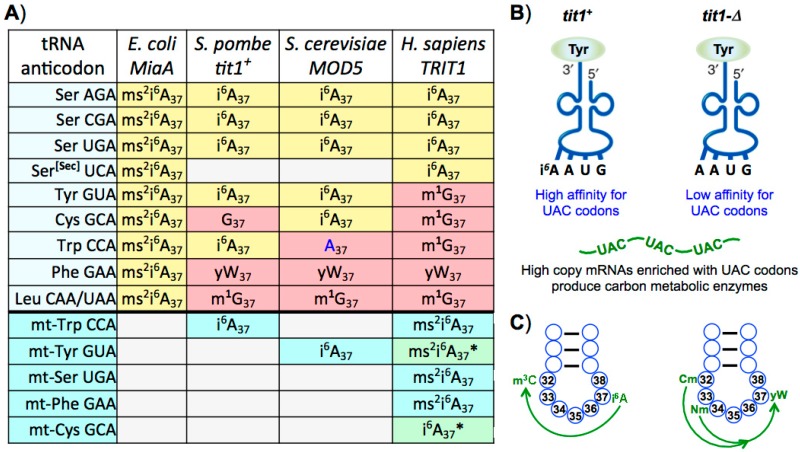
Species-plasticity in i^6^A37-associated anticodons, potential for secondary code information use and position 37 modification interdependence. (**A**) Summary of i^6^A_37_-associated anticodons in four species. The gene names for the tRNA isopentenyltransferases that form i^6^A_37_ are provided under the species names in the top row. The anticodons above the thick horizontal are for cytosolic tRNAs and below are mitochondrial tRNAs; asterisks reflect data from bovine not human; data are from [127,129,130,131]; (**B**) Summary of effect of i^6^A_37_ on decoding activity and translation by tRNA^Tyr^GAU in *Shizosaccharomyces pombe*, depicted as a cartoon (see [102,132]); (**C**) Crosstalk or interdependence among modifications of position 37 nucleotide and other modifications in the anticodon loop of eukaryotic tRNAs. Left panel: m^3^C_32_ is dependent on i^6^A_37_ [133]. Right panel: dependence of yW37 on the ribose methylations at positions 32 and 34 [134,135]. *E. coli*: *Escherichia coli; S. pombe*: *Shizosaccharomyces pombe; S. cerevisiae*: *Saccharomyces cerevisiae*; *H. sapiens*: *Homo sapiens*.

In stark contrast to the omnipresence of t^6^A_37_ on tRNAs that read ANN codons (N = U, C, G or A), is the variability of i^6^A_37_ among eukaryotes [127]. tRNAs with i^6^A_37_ are also found in all domains of life, and are indeed limited to those that read UNN codons, but occurrence of i^6^A_37_ on specific anticodons varies in eukaryotes and even among different species of yeast. In bacteria, i^6^A_37_ and its hypermodified forms hereafter referred to collectively for simplicity as i^6^A_37_, are found on all tRNAs that decode the 13 sense UNN codons (N = U, C, G or A) (14 including selenocysteine (SerSec) UGA): those that decode the Trp codon, the two codons each for Cys, Tyr, and Phe, as well as the four of six codons for Ser and two of six codons for Leu (and the UGA codon for SerSec). However, i^6^A_37_ is limited to variably different subsets of these (cytosolic) tRNAs in eukaryotes. It is excluded from tRNAs^Leu^ and tRNAs^Phe^, the former of which contains m^1^G_37_ (1-methyl-G) and the latter contains a bulky hypermodified G known as wybutosine at 37 (yW37). Furthermore, i^6^A_37_ is absent on tRNAs for Cys, Tyr and Trp in a species-specific manner such that budding yeast, fission yeast and human cells each contain distinct subsets of i^6^A37-containing tRNAs [127]; in cases where these tRNAs lack i^6^A_37_ they usually have an encoded G at 37, found as m^1^G in the tRNA [128,129] (Figure 5A). Again, this variability is in stark contrast to the omnipresence of t^6^A_37_ on tRNAs that read ANN codons (see [101,123,126]).

Species-specific patterns of i^6^A_37_ distribution on different subsets of tRNAs is consistent with potential for use a part of a secondary genetic code information system. Evidence that some i^6^A37-modified tRNAs may be keyed to expression of specific mRNAs of related function in a cognate codon-dependent manner can be found in bacteria and yeast. Of the six Leu codons, two begin with U, UUG and UUA (UUX hereafter) accounting for about 25% of Leu codons in *E. coli*. The mRNAs encoding the stress response transcription factor σ, RpoS/σ38 and a positive regulator of its stability, IraP are enriched in Leu-UUX codons which sensitize their expression to loss of MiaA, the bacterial tRNA isopentenyltransferase [136,137]. Synonymous codon swaps of UUX-Leu to CUX-Leu within *RpoS* and *IraP* suppress the effects of MiaA deletion on their translation, providing evidence that i^6^A_37_ enhances decoding of UUX-Leu codons [136].

In the fission yeast, *S. pombe*, five cytosolic tRNAs contain i^6^A_37_, three that decode four of the six Ser codons (UCN), one for the two Tyr codons (UAU and UAC) and one for the Trp codon (UGG) [127]. By comparing β-galactosidase reporters that bear codon swaps of Tyr codon 503 which is required for efficient catalytic activity, in wild-type and strains deleted of the *S. pombe* tRNA isopentenyltransferase, it was estimated that i^6^A_37_ enhances the ability of tRNA^Tyr^GUA to decode its cognate codon UAC by 3–4 fold [132]. Tyr codons in *S. pombe* are differentially distributed such that the ratio of UAC-to-UAU is nearly 4.3-to-1 in abundant mRNAs that encode carbon metabolizing energy enzymes but is 0.54-to-1 in low abundance mRNAs, a nearly eight-fold enrichment of the codon with C in the third position in the highly-expressed mRNAs [102]. It was shown that absence of i^6^A_37_ specifically on cytosolic tRNA^Tyr^GUA leads to the carbon source-specific growth deficiency phenotype of fission yeast lacking the tRNA isopenyltransferase, Tit1 [102]. Overexpression of cytosolic tRNA^Tyr^GUA in *tit1-*deletion cells rescues the carbon-specific growth deficiency [102] (schematized in Figure 5B).

*S. cerevisiae* has i^6^A_37_ on cytosolic tRNA^Ser^, tRNA^Tyr^ and tRNA^Cys^ whereas *S. pombe* has i^6^A_37_ on cytosolic tRNA^Ser^, tRNA^Tyr^ and tRNA^Trp^ [127], consistent with database entries. By contrast to each of these yeasts, the cytosolic i^6^A37-tRNAs of human cells are limited to tRNA^Ser^ and tRNA^Ser[Sec]^ which carries selenocysteine to UGA codons [130]. By contrast to the species-variable need for i^6^A_37_, there is almost no variability in t^6^A_37_, presumably reflecting species-specific sensitivity to the context nature of the codon:anticodon A:U vs. U:A base pairs. By limiting the number of tRNAs that carry i^6^A_37_ and grouping them in distinct subsets (in different species) might enhance discrete control of the subsets of cognate responsive mRNAs.

That eukaryotes have distributed i^6^A_37_ among smaller subsets of tRNAs whereas bacteria have a wide distribution of i^6^A_37_ and still appear to manage its use for secondary genetic code information suggests that the same may be applicable to t^6^A_37_. In this regard, we note that bacteria appear to use i^6^A_37_ for secondary code information in mRNAs enriched in two codons of a six box (Leu) codon set. Thus, analogous opportunity might be available to the two-codon Ser and Arg t^6^A_37_-containing tRNAs.

## 16. Interdependence of Position 37 Modifications in Eukaryal tRNAs

While some tRNA modification enzymes work as a single polypeptide, several operate as a two-subunit heteromeric complex comprised of the products of different genes [138]. For some chemically-complex modifications of anticodon loop nucleotides, the sequential actions of multiple enzyme activities are required (see [2,139]). Several lines of evidence indicate that some modification activities are dependent on a modification elsewhere on the tRNA. An early example was Queuosine (Q) formation in the anticodon wobble position of marsupial mitochondrial-tRNA^Asp^ which occurs only after C to U editing at the second anticodon position [140]. 7-methyl-G46 (m^7^G_46_) positively affects Gm18 and m^1^G_37_ modifications in *Thermus thermophilus* [141] whereas formation of pseudouridine at position 55 negatively affects Gm18, m^5^s^2^U_54_ and m^1^A_58_ modifications [142]. In *Trypanosoma brucei* cytosolic tRNA^Thr^ editing of C32 to U32 stimulates the efficiency of A34I editing [143]. It was also documented that m^5^C_38_ modification by the DNMT2 homologs, Pmt1 and DmnA in *S. pombe* and *D. discoideum,* respectively, depends on prior Q modification of position 34 and this is stimulated by queuine in the media [144].

We highlight two recent cases that involve position 37 of different tRNAs, the yW37 and i^6^A_37_ of tRNA^Phe^ and tRNAs^Ser^, respectively and their interdependent modifications elsewhere in their anticodon stem loop (ASL) [133,134] (Figure 5C). Regarding the position 37 modifications of these tRNAs, while bacterial tRNAs^Phe^ and other bacterial tRNAs that decode UNN codons contain i^6^A_37_, the tRNAs^Phe^ in eukaryotes almost without exception contain the hypermodified G nucleotide, yW37. For the latter, 2′-*O*-methylribose modification of C32 and N34 by Trm7/Trm732 and Trm7/Trm734 respectively are required for efficient conversion of m^1^G_37_ to yW37 of yeast tRNA^Phe^, a complex modification codependency that is also found for human tRNA^Phe^ [134]. Moreover, mutations in various alleles of FTSJ1, the human *TRM7* homolog in this activity circuit cause/are associated with a range of developmental disorders [135].

For a subset of tRNAs^Ser^, the interdependency of positions 37 and 32 is such that i^6^A_37_ is prerequisite for formation of m^3^C_32_ [133], the latter of which is a eukaryote-specific tRNA modification. It was noted that the i^6^A37-m^3^C_32_ ASL modification circuit may have implications for disease as mutations in TRIT1, the gene responsible for i^6^A_37_ formation on cytosolic and mitochondrial tRNAs, cause human pathology due in large part to mitochondrial dysfunction [145,146]. In humans i^6^A_37_ is found on cytosolic tRNAs^Ser^ and tRNA^Ser[Sec]^ in addition to several mitochondrial tRNAs that contain i^6^A_37_ or ms^2^i^6^A_37_ among which is the major species tRNA^Ser(UGA)^ [130] that also contains m^3^C_32_ (also see [102,131]). Although yeast *S. cerevisiae* and *S. pombe* mutants lacking m^3^C_32_ exhibit no growth phenotype under various conditions [133,147], the *S. cerevisiae* double mutant *trm140Δ trm1Δ* that also lacks m^2^_2_G_26_ on multiple overlapping tRNAs exhibits slow growth in the presence of the translation inhibitor cycloheximide [147].

Whereas formation of yW37 on tRNA^Phe^ requires modification activity at positions 32 and 34, for tRNAs^Ser^ the prerequisite order would appear to be opposite as the m^3^C_32_ activity requires preexisting i^6^A_37_ (Figure 5C). We emphasize that m^3^C_32_ and yW37 are eukaryote-specific. Thus, it might appear that as tRNA^Phe^ was shifted from the domain of i^6^A_37_ in bacteria to yW37 in eukaryotes the latter modifications became dependent on C32/N34 modification activities.

It was interesting that m^3^C_32_ modification occurs in the absence of i^6^A_37_ on tRNAs^Thr^ and the tRNA^Ser^GCU that is not modified with i^6^A_37_ [133] but are instead modified with t^6^A_37_. This suggested that the t^6^A_37_ found on the tRNAs^Thr^ and tRNA^Ser^GCU, might be required for m^3^C_32_ formation [133]. Dependency of m^3^C_32_ on t^6^A_37_ was indeed reported for tRNAs^Thr^ in *S. cerevisiae* [148] (also see below). The cumulative observations suggest that position 37 modifications have evolved in eukaryotes as part of interdependent circuits. For the eukaryotic tRNAs^Ser^ and tRNAs^Thr^ with i^6^A_37_ or t^6^A_37_, their eukaryote-specific m^3^C_32_ modification activity became dependent on them. In higher eukaryotes m^3^C_32_ is also found on tRNAs^Arg^YCT which also carry t^6^A_37_, suggesting the possibility of similar codependence [133].

## 17. Amplification and Diversification of Eukaryal tRNA Methyltransferases

Methylation is a most pervasive and ancient modification to tRNA, occurring on all of the bases in all domains of life, in some cases at multiple positions on the base, and/or in conjunction with or added to other modifications, as well as to the ribose moiety of the nucleotide [149]. The tRNA methyltransferases (TRMs) encompass a diverse family of enzymes, that includes several different structural classes and mechanisms of catalysis, that appear to have evolved independently [149]. A compilation of mammalian homologs of the known *S. cerevisiae* TRMs revealed multiple for several [150]. As alluded to above and detailed below, an interesting TRM gene amplification occurred in fission yeasts whose study revealed functional subspecification of ASL substrates and dependence on A37 modification [133]. We therefore did a search for amino acid sequence homologs of *S. cerevisiae* tRNA modification enzyme genes and included *S. pombe*, mouse and human homologs (excluding genes for the large multisubunit complexes elongator and KEOPS/EKC) (Table 2). From this it would appear that gene duplications and amplifications were limited to the TRMs (Table 2, *S. cerevisiae* genes in bold font). Genes for eight of the 16 TRMs in *S. cerevisiae* appear to have duplicated copies in at least two of the other species, TRMs 1, 2, 4, 61, 7, 9, 10 and 140.

Notably, of the six TRMs that modify a nucleotide in the anticodon loop (indicated by asterisks in Table 2), four have been subjected to gene amplifications in at least two of the other species, TRMs 4, 7, 9 and 140, and two were duplicated in all three species, TRMs 4 and 140. It is also noteworthy in this regard that several of the TRMs, especially TRM4 are required for normal programmed translational response to stress [115,116,120]. TRMs 4 and 140 were apparently subjected to duplication in *S. pombe*, the latter as *trm140^+^* and *trm141^+^* which have been adapted for isoacceptor-specific substrate activity (below). Systematic examination of *S. cerevisiae* tRNA modification mutants’ response to a panel of stress conditions was performed before the discovery of TRM140 [115], although some evidence suggests a translational stress in *trm140-deletion* mutants [147].

As new TRMs arose by gene duplication and diversification they might have adopted specificity for a subset of the substrates previously modified by a single enzyme in *S. cerevisiae*, or in some cases they may have diversified and adopted new substrates. We believe that there is some evidence for both scenarios.

Analysis of *S. pombe* sequence homologs of the product of the *S. cerevisiae TRM140* gene, which is responsible for m^3^C_32_ modification of tRNAs^Ser^ and tRNAs^Thr^ provides an example of adopting specificity for a subset of substrates [133]. In *S. cerevisiae*, *TRM140* alone modifies both tRNAs^Ser^ and tRNAs^Thr^ [147,152]. However, the *S. pombe* sequence homologs *trm140^+^* and *trm141^+^* exhibit distinct substrate specificity: *trm140^+^* for the three tRNAs^Thr^, and *trm141^+^* for the four tRNAs^Ser^ [133]. This provides evidence for a tRNA modification enzyme gene duplication and diversification in which the resulting paralogs each adopted specificity for a subset of the substrates modified by a single enzyme in *S. cerevisiae*. The course of evolutionary adaptation in this case is particularly interesting as it would appear to be reflective that the single enzyme in *S. cerevisiae*, Trm140 is actually bifunctional as it uses two distinct modes of recognition to modify tRNAs^Ser^ and tRNAs^Thr^ [148]. For tRNAs^Thr^, *S. cerevisiae* Trm140 relies on the presence of their shared anticodon loop motif, G35-U36-t^6^A_37_ as a recognition determinant, whereas for tRNAs^Ser^, Trm140 relies on their common large variable loop and i^6^A_37_ as determinants and on its interaction with the seryl-tRNA synthetase [148]. From this one might expect that *trm140^+^* and *trm141^+^* may each employ one of the two distinct recognition modes used by *TRM140* although this remains to be determined. Notable is that while, tRNA^Ser^IGA is not found to contain m^3^C_32_ in *S. cerevisiae* in which it contains pseudouridine-32, tRNA^Ser^IGA of *S. pombe* and higher eukaryotes contains m^3^C_32_ [129]. Although mild slow growth of *S. cerevisiae* double mutant *trm140Δ trm1Δ* in the presence of cycloheximide might suggest activity related to translocation of the ribosome during mRNA translation [147], the function of this eukaryote-specific modification remains unknown. It should be noted that m^3^C_32_ results from endocyclic nitrogen methylation that results in a +1 charge to the nucleotide (see [149]).

With regard to evolutionary directionality of the gene changes involved, deeper analysis might determine if the *S. cerevisiae TRM140* may represent the ancestral eukaryotic tRNA m^3^C_32_ enzyme or if two specialized forms arose by another evolutionary pathway and somehow merged in the budding yeasts to form *TRM140*. It is interesting to note that *TRM140* is indeed an unusual gene, also known as *ABP140* which is a fusion of an actin-binding domain connected to the coding sequence for the Trm140 modification enzyme, whereas in other species, the Trm140 domain stands alone [153]. Intriguingly, the two domains in *ABP140* are separated by a +1 frameshift signal in the ABP140/TRM140 mRNA. *ABP140/TRM140* is one of two genes in *S. cerevisiae* that contain a +1 frameshift signal in their mRNA (the other is *EST3*), which for *TRM140* resides upstream of the tRNA modifying domain [153]. Trm140 activity is produced from the zero frame, in the absence of +1 frameshifting [152]. It has been noted that an abundance of Ser and Thr codons reside upstream of the +1 frameshift signal [147]. It is also intriguing that +1 frameshifting in the *EST3 and ABP140* mRNAs is controlled by the activities of tRNA^Ser^GCU and tRNA^Arg^CCU, respectively, which must decode the second codon in their +1 slippery frameshift signals [153]. It is therefore noteworthy that tRNA^Ser^GCU is an efficient substrate of Trm140 in *S. cerevisiae* [148], whereas tRNA^Arg^CCU is not known to be but in human and mouse is one of two tRNAs other than Thr and Ser that carry m^3^C_32_ where it is as highly efficiently modified as are the tRNAs^Thr^ [133] and also carries two apparent Trm140 specificity determinants, U36 and t^6^A_37_ [148]. It is therefore plausible that overexpression of Trm140 might drive m^3^C_32_ formation on tRNA^Arg^CCU and promote +1 frameshifting with feedback down regulation of Trm140 activity.

Humans have four predicted sequence homologs of Trm140: encoded by METTL2A, METTL2B, METTL6 and METTL8 [133,150]. The 2A and 2B homologs are very similar to each other and show highest homology to *S. pombe trm140+* while L6 and L8 show higher homology to *trm141^+^* [133]. Knock-down of METTL2B decreased the m^3^C levels in human cells, apparently by ≥50%, presumably reflecting most of total m^3^C_32_ [152]. As alluded to above, apart from tRNAs^Ser^ and tRNAs^Thr^, two human tRNAs^Arg^ isotypes also carry m^3^C_32_ while a minor subset of tRNAs^Ser^ carry m^3^C in the variable loop [128,129,133]. Because gene duplication and substrate subset specificity is a precedent for the *TRM140*/*trm140^+^*/*trm141^+^* system, it is reasonable to suspect that the even greater expansion of *TRM140*-homologs in mammals might have driven a wider distribution of m^3^C_32_ in tRNAs as compared to yeast, both in the additional substrates, tRNAs^Arg^, and at a different position, the variable loop, of a subset of the original substrates, the tRNAs^Ser^, although these speculations remain to be tested.

Two methyltransferase genes that were expanded in different species are those which modify more than one position. *S. cerevisiae* Trm4 is responsible for 5-methylcytidine (m^5^C) on at least four different positions (C34, C40, C48 and C49) on different tRNAs [154]. There are two homologs in *S. pombe* (SPAC17D4.04 and SPAC23C4.17) and 6 homologs in humans (NSUN1-6) (Table 2 and [150]). NSUN2 is responsible for m^5^C at positions 34, 48, 49 and 50 [155] while NSUN6 adds m^5^C at position 72 [156], which is not observed in *S. cerevisiae*. The second TRM gene that is single copy in *S. cerevisiae* but amplified in other eukaryotes is Trm7, responsible for ribose methylation at C32, C34 and G34 of different tRNAs [157]. In humans, there are three homologs for this enzyme: FTSJ1, FTSJ2 and FTSJ3 [150].

Mutations in FTSJ1 are associated with mental retardation [135] and mutations in NSUN2 with intellectual disability as well as cardiac diseases [155,158], indicating their importance in human development and health. As these genes are identified solely based on homology and their potential substrate specificities are not known, we cannot rule out that some may modify non-tRNA substrates. Some data indicate that FTSJ2 and FTSJ3 may be involved in modification of mitochondrial and cytoplasmic rRNA, respectively [159,160].

Other considerations regarding enzyme duplications should be reflected. First, some species have two genes encoding proteins that modify or process cytosolic or mitochondrial tRNAs differentially, and others use specific isoforms of a single gene product to do so, although there may be no readily apparent evolutionary consistency of the patterns. For example, in most eukaryotes including *S. cerevisiae, Drosophila* and humans, one tRNase Z (L–refers to long form) gene encodes both nuclear and mitochondrial forms of tRNase Z (L), the enzyme that cleaves the trailer sequences from the 3′ ends of tRNA precursors, whereas (all four) *Schizosaccharomyces* species contain two essential tRNase Z (L) genes whose products are targeted either to the nucleus or mitochondria [161]. Thus, gene duplications may account for some expansions of tRNA associated enzymes in isolated lineages or species, but with no apparent consistency predictive of outcome without functional studies.

A second consideration of deciphering amplifications of enzyme homologs in multicellular organisms is one of tissue or temporal specificity. In this case a second homolog may increase global tRNA modifications in a tissue- or time-specific manner, e.g., to aid development, or perhaps associated with a tissue-specific tRNA. Although some tRNAs are expressed in a tissue-specific manner, these have been associated with shifts in the total pool [162], whereas in some cases individual tRNAs have been documented to be restricted to the central nervous system and are known to be important determinants of development [14,163]. We are unaware of a specific link between a tissue-specific modification and a tissue-specific tRNA. Thus, in the case of the TRMs in which there is precedent for the evolutionary acquisition of new tRNA position-specific methylations, a gene duplication should not necessarily suggest recruitment of a new substrate, as it may reflect tissue-specific, organelle-specific or temporal-specific isoform with activity for the same substrate(s). 

In the yeasts *S. cerevisiae* and *S. pombe*, a single enzyme, Trm10, is responsible for creation of the universally conserved 1-methyl-G at position 9 (m^1^G_9_) [164]. In humans, there are three homologs for *TRM10*: TRMT10A, TRMT10B and TRMT10C [165]. All three possess m^1^G_9_ modification activity [165]. Of these, TRMT10C localizes to mitochondria and modifies mitochondrial tRNAs while TRMT10A and TRMT10B modify cytoplasmic tRNAs [165]. In accordance with its localized function, mutations in TRMT10C cause mitochondrial disorders [166]. Association of mutations in TRMT10A with microcephaly and young onset diabetes reaffirms its regulatory role in lineage differentiation [167,168,169,170,171]. Basal levels of TRMT10A protein expression is observed in all tissues but is enriched in brain and pancreatic β cells accounting for the tissue-specific developmental disorders [167].

Another interesting feature of tRNA modification enzyme evolution is DNMT2 which forms m^5^C_38_ in the anticodon loops of tRNAs^Asp^GUC [172]. DNMT2 is a member of the DNMT (DNA methyltransferase) family of proteins [172], other members of which are DNMT1 and DNMT3, discovered to form m^5^C at CpG sites as repressive and epigenetic marks in genomic DNA of higher eukaryotes [173]. DNMT2 also forms m^5^C_38_ in multiple tRNAs in a range of species including a prokaryote (reviewed in [174]). This modification can protect against endonucleolytic cleavage within the anticodon during stress [175,176], and potentially regulate formation of tRNA fragments (tRFs). Mutations in DNMT2 that alter its modification activity have been observed in cancers suggesting a role in tumorigenesis [177]. The phylogenetic homology of the DNMT family members and presence of a DNMT2 tRNA-modifying enzyme in a prokaryote suggest it as ancestral of the DNMT1 and DNMT3 activities involved in regulating genome biology in higher eukaryotes. It is intriguing in this regard that DNMT2 can more efficiently form m^5^C on a deoxynucleotide in the context of a tRNA than on a ribonucleotide at the same position, and was used to engineer a guide RNA to direct DNMT2 to methylate DNA [174].

## 18. The tRNAs in Health and Disease

As noted above, tRNA anticodon modifications have been keyed to codon use and stress responses in bacteria and eukaryotes, and these include oxidative stress and dealing with hypoxia. Although such connections have not come to light for cytosolic tRNAs in higher eukaryotes we might expect that similar pathways may be beneficial to health during stress responses, including to oxidative stress.

Certainly, mutations in a number of the single tRNA sequences encoded in the mitochondrial DNA have been associated with human pathology. These mutations impair translation of mitochondrial DNA-encoded mRNAs by mitochondrial ribosomes and the production of ATP via oxidative phosphorylation (for excellent comprehensive review see [178,179]). There are also numerous mitochondrial-disease conditions due to mutations in nuclear genes that encode proteins that are transported to mitochondria, such as tRNA processing and modification enzymes, and tRNA synthetases [178,179].

The subject of hereditary mutations in tRNA-associated synthetases, processing and modification enzymes that impair the function of nuclear-encoded cytosolic tRNAs has also been reviewed from various perspectives [158,180,181]. In several of these cases, subsets of cytosolic tRNAs of the same and/or multiple isoacceptor families are compromised.

Because the nuclear genes encoding each of the ca. 47 tRNA anticodon families in mammals are multicopy, mutation to an individual tRNA gene might not be expected to be pathologic. However, this perspective was overturned upon report of a mutation in a central nervous system-specific tRNA^Arg^UCU gene that is associated with widespread neurodegeneration in mouse [14,163]. This reflects a highly specific association between a defective tRNA and neuropathology but adds to what appears to be a more general association between defects in tRNA biogenesis–metabolism modifications and neurodevelopmental disorders [36]. For example, a multitude of heritable mutations to four different cytoplasmic tRNA synthetases (tyrosyl, lysyl, glycyl, alanyl) cause Charcot–Marie–Tooth disease and related neuropathologies [182]. In this case, translational errors caused by mischarging due to faulty editing or other synthetase deficiencies may sensitize neurons to dysfunction [183]. In addition to mutations to tRNA processing and modification enzymes, a large number of mutations have been cataloged to several genes involved in tRNA transcription that also lead to neurodevelopmental disorders (reviewed in [85]).

The single gene Mendelian model of disease clearly applies to the disorders caused by mutations in tRNA-associated enzymes as well as mutation to the central nervous system (CNS)-specific tRNA^Arg^UCU gene itself that is associated with neurodegeneration [14,163]. However, there is another view of how disturbances of tRNA pool homeostasis may influence common disorders for which there are genetic influences, namely, as a disease modifier.

Genetic variances among individuals in any of the numerous enzymes that affect tRNA metabolism including modification and charging activities, can alter the relative activity balance of the tRNA pool. Such imbalances in tRNA pool activity, even very subtle, could plausibly cause ribosome pausing or other shifts in translation to the most sensitive mRNAs in the transcriptome, an outcome of which could cause polypeptide misfolding-related proteopathy. The individual mRNAs in a complex population vary in their codon use and some exhibit greater tolerance for translational error than others, referred to as ‘translational robustness’ [184]. While mRNA codon use and tRNA supply are thought to match in healthy tissue, subtle offsets might have significant consequences. Depending on the difference in the tRNA pool from ‘normal’ in any particular limiting tRNA condition and the hierarchal translational robustness of the mRNAs in the cell type at hand, different phenotypes may emerge in context-dependent manner. Tissues with major products of central importance such as exocrine pancreas may be highly susceptible to protein misfolding [185]. Indeed, deficiency in the tRNA anticodon wobble modification enzyme, CDKAL1, was first linked to type 2 diabetes by genome-wide association studies through population genetics [186,187]. Codon use in the brain-specific genes has been unusually conserved in mammals [188], fitting with the idea that the CNS may be especially sensitive to tRNA pool balance, possibly reflecting vulnerability to perturbance of proteostasis accounting for susceptibility to disorders of tRNA metabolism.

This view appreciates tRNA genes in higher eukaryotes as heritable units of trait, as clearly documented by the work of Ackerman and colleagues [14]. However, trait effects are likely not limited to single tRNA genes. Even among individual humans, tRNA gene copy number is quite variable [24,25], as clusters of some tRNA genes can differ in copy number and other single tRNA gene loci can be found in homozygous, heterozygous or nullizygous form [24]. Although the cellular tRNA pool is principally determined by the tRNAome, its composition will also be influenced by the collective efficacy of the tRNA-associated processing, modification and charging activities. The ultimate readout for which is their transcriptome. Relative match between the tRNA and mRNA pools determines phenotypes. From this view one can see how a disorder associated with a well characterized single gene mutation may appear to be unpredictably modified in different ‘patients’ including with variable penetrance. It stands to reason that tRNAs comprise a significant component of human variability and deciphering this contribution to our overall genetic difference is a challenge for the future of medicine and genetics more generally.

## 19. Conclusions

Features that accompanied the emergence of free living Eukarya were high numbers and sequence diversity of tRNA genes relative to the Bacteria and Archaea. This was associated with reorganization of tRNA genes as individual monocistronic transcription units under the exclusive control of one of the three eukaryote-specific RNA polymerases, RNAP III. Reorganization of tRNA genes from polycistronic together with other RNA types in bacteria and archaea, to monocistronic under the control of a separate RNAP also empowers them as individual genetic units. The transcription termination mechanism of RNAP III attaches a 3′ oligo(U) motif to all of its nascent pre-tRNA transcripts which directly targets them to the eukaryote-specific, pre-tRNA chaperone, La protein. As chaperones can be evolutionary drivers because they can buffer mutations in their substrates and thereby lead to emergence of new functional genes from cryptic alleles [83,84], it would seem that La may have served such a function for tRNA genes. Thus, tRNA gene amplification and diversification coupled to a chaperone system that also includes nuclear modification enzymes would plausibly create and establish otherwise susceptible tRNA sequences for trial and selection.

An evolutionary upsurgence of tRNA genes encoding A34 anticodons occurred in eukaryotes along with diversified use of the A34 to I34 modification and a cognate shift in codon use. This was associated with a conserved exclusion of G34 anticodon tRNA genes for the same amino acid, suggestive of the establishment of a regulatory system that makes use of the redundancy component of the genetic code. Ample biological evidence from yeast and bacteria involving other wobble modifications provide evidence that these and the i^6^A_37_ modification contribute to a system for programmable tunable translation of groups of cognate codon-biased mRNAs under different stress conditions.

The size of eukaryotic tRNAomes generally increase with species complexity and possibly developmental challenges as several mammals, fish and other vertebrates have several to tens of thousands of predicted tRNA genes. In the large tRNAomes of some mammals and other vertebrates are included up to ca. 6% of tRNA genes with anticodons that appear to have been systematically excluded from all other eukaryotic tRNAomes, in some cases because they might cause wobble miscoding, raising questions about the significance of their existence, expression, modification and function.

Some tRNA modifications in the ASL are dependent on other ASL modifications. Modification of position 37 is important for mRNA decoding and i^6^A_37_ exhibits species-specific plasticity in the identities of associated tRNAs among distant and parallel species, and has been linked to cognate codon-biased mRNA translation in bacteria and yeast. Eukaryotic tRNA methyltransferases were amplified and diversified, exemplified by TRM140 which modifies two tRNA isoacceptors in one yeast but in another yeast each of two paralogs modifies one or the other isoacceptor type. Other amplified TRMs modify ASLs involved in stress responses. A perspective that emerges is that eukaryotic tRNAomes expanded in ways that supported programmed tunable translation of cognate codon biased mRNAs and disparate use of the redundancy (synonymous) component of the genetic code.

## Figures and Tables

**Table 1:** Results of gene ontology (GO) analysis of *S. pombe* mRNAs sorted by ratio of UCU to UCC codons after sorting by mRNA copy number (see text). The GO results for the top 41, middle 41 and bottom 41 mRNAs are shown (see text). GeneOntology Consortium. Enrichment analysis using PANTHER. #A: number of mRNAs in the GO category; #B: number of mRNAs in the test group.

	GO Molecular Function Complete	#A	#B	Expected	Fold Enrichment	*p*-Value (Only *p* < 0.05 Shown)
**Top 41** **Score 1.0** **All UCU, no UCC**	rRNA binding	38	5	0.33	15.37	1.33 × 10^−2^
	Structural constitutent of ribosome	213	24	1.82	13.16	3.62 × 10^−19^
	Structural molecule activity	246	25	2.11	11.87	4.05 × 10^−19^
**Middle 41** **Score 0.5** **UCU = UCC**	Unclassified	72	3	0.59	5.08	0.00
**Bottom 41** **Score 0** **All UCC, no UCU**	Unclassified	74	1	0.59	1.69	0.00

**Table 2:** Sequence homologs of *S. cerevisiae* tRNA modification enzymes in *S. pombe*, mouse and human. Homologs of enzymes listed in [36] were identified by BLASTp analysis [151].

*Saccharomyces cerevisiae*	*Schizosaccharomyces pombe*	*Homo sapiens*	*Mus musculus*	Modification
CCA1	cca1+	CCA1	CCA1	CCA
DUS1	dus1+	DUS1L	DUS1L	D (16 and 17)
DUS2	dus2+	DUS2	DUS2	D (20)
DUS3	dus3+	DUS3L	DUS3L	D (47)
DUS4	dus4+	DUS4L	DUS4	D (20a and 20b)
MOD5 *	tit1+	TRIT1	TRIT1	i^6^A (37)
PUS1 *	pus1+	PUS1	PUS1	Ψ (26–28, 34–36, 65, 67) **
PUS3/DEG1 *	deg1+	PUS1	PUS1	Ψ (38, 39)
PUS4	pus4+ (predicted)	PUS4 (predicted)	PUS4 (predicted)	Ψ (55)
PUS6	SPCC4G3.16	RPUSD2	RPUSD2	Ψ (31)
PUS8 *	SPAC18B11.02c	RPUSD4	RPUSD4	Ψ (32)
PUS7 *	SPBC1A4.09	PUS7	PUS7	Ψ (13, 35)
RIT1	$\underset{¯}{\text{SPAC3F10.06c}}$			Ar(P) (64)
TAD1 *	SPBC16A3.06	ADARB1/ADAR2	ADARB1/ADAR2	I (37)
TAD2 *	*tad2+*	ADAT2	ADAT2	I (34)
TAD3 *	*tad3+*	ADAT3	ADAT3	
TAN1	SPBC25H2.10c	THUMPD1	THUMPD1	ac^4^C (12)
Kre33	SPAC20G8.09c	NAT10	NAT10	
THG1	*thg1+*	THG1L	THG1L	G (-1)
**TRM1**	*trm1+*	TRMT1 TRMT1L	TRMT1 TRMT1L	m^2^_2_G_26_
**TRM2**	*trm2+*	TRMT2A TRMT2B	TRMT2A TRMT2B	m5U (54)
TRM3		TARBP1	TARBP1	Gm (18)
TRM4 *	SPAC17D4.04 SPAC23C4.17	NSUN2 NOP2/NSUN1 NSUN5 NSUN4 NSUN6 NSUN3	NSUN2 NOP2/NSUN1 NSUN5 NSUN4 NSUN6 NSUN3	m^5^C (C34, C40, C48 and C49) **
TRMT5 *	*trm5+*	TRMT5	TRMT5	m^1^G (37)
TRM6	*gcd10+*	TRMT6	TRMT6	m^1^A (58)
**TRM61**	*cpd1+*	TRMT61A TRMT61B	TRMT61A TRMT61B	
TRM7 *	*trm7+*	FTSJ1 FTSJ2 FTSJ3	FTSJ1 FTSJ2 FTSJ3	Cm (32) & Nm (34) ***
TRM8	*trm8+*	METTL1	METTL1	m^7^G (46)
TRM9 *	*trm9+*	KIAA1456/Trm9L ALKBH8	KIAA1456/Trm9L ALKBH8	mcm^5^U (34)
**TRM10**	*trm10+*	TRMT10A TRMT10B TRMT10C	TRMT10A TRMT10B TRMT10C	m^1^G (9)
TRM11	*trm11+*	TRMT11	TRMT11	m^2^G (10)
TRM12 *	*trm12+*	TRMT12	TRMT12	Wyosine (37)
TRM13	*trm13+*	TRMT13	TRMT13	Nm (4) ***
TRM44	SPCC663.10	TRMT44	TRMT44	Um (44)
TRM140 *	*trm140+* *trm141+*	METTL2 METTL6 METTL8	METTL2 METTL2B METTL6 METTL8	m^3^C (32)
TYW1 *	*tyw1+*	TYW1	TYW1	yW
TYW3 *	*tyw3+*	TYW3	TYW3	
TYW4 *	*tyw4+*	TYW4	TYW4	

* Nucleotides modified reside in the anticodon loop. ** Numbers given are for *S. cerevisiae*. It may vary for another species (see page 16). *** N: any nucleotide. **Bold** font reflects genes with apparent duplications. D: dihydrouridine; Ψ: pseudouridine; Ar(P): 2′-*O*-ribosyladenosine (phosphate); i^6^A: *N^6^*-isopentenyladenosine; I: inosine; ac^4^C: *N^4^*-acetylcytidine; G: guanosine; m^2^_2_G26: *N^2^ N^2^*-dimethylguanosine; m^5^U: 5-methyluridine; Gm: 2′-*O*-methylguanosine; m^5^C: 5-methylcytidine; m^1^G: 1-methylguanosine; m^1^A: 1-methyladenosine; Cm: 2′-*O*-methylcytidine; Nm: 2′-*O*-methylnucleoside (N can be any nucleotide); m^7^G: 7-methylguanosine; mcm^5^U: 5-methoxycarbonylmethyluridine; m^2^G: *N^2^*-methylguanosine; Um: 2′-*O*-methyluridine; m^3^C: 3-methylcytidine; yW: wybutosine. List of modifications and the *S. cerevisiae* enzymes except for m^3^C are adapted from [35] (for m^3^C enzyme nomenclature see [133]).
